# Parkinson Network Eastern Saxony (PANOS): Reaching Consensus for a Regional Intersectoral Integrated Care Concept for Patients with Parkinson’s Disease in the Region of Eastern Saxony, Germany

**DOI:** 10.3390/jcm9092906

**Published:** 2020-09-08

**Authors:** Kai F. Loewenbrück, Doron B. Stein, Volker E. Amelung, Robert Bitterlich, Martin Brumme, Björn Falkenburger, Annekathrin Fehre, Tim Feige, Anika Frank, Carola Gißke, Claudia Helmert, Linda Kerkemeyer, Andreas Knapp, Caroline Lang, Annegret Leuner, Carina Lummer, Mirella M. N. Minkman, Gabriele Müller, Marlena van Munster, Hannes Schlieter, Peter Themann, Nick Zonneveld, Martin Wolz

**Affiliations:** 1 Department of Neurology, University Hospital Dresden, 01307 Dresden, Germany; Robert.Bitterlich@uniklinikum-dresden.de (R.B.); Bjoern.Falkenburger@uniklinikum-dresden.de (B.F.); Tim.Feige@uniklinikum-dresden.de (T.F.); anika.frank@uniklinikum-dresden.de (A.F.); 2 Clinical Trial Unit, German Center for Neurodegenerative Diseases (DZNE) Dresden, 01307 Dresden, Germany; 3 Institute for Applied Health Services Research (inav), Schiffbauerdamm 12, 10117 Berlin, Germany; stein@inav-berlin.de (D.B.S.); amelung@inav-berlin.de (V.E.A.); brumme@inav-berlin.de (M.B.); kerkemeyer@inav-berlin.de (L.K.); lummer@inav-berlin.de (C.L.); 4 Department of Neurology, Elblandklinikum Meißen, 01662 Meißen, Germany; Annekathrin.Fehre@elblandkliniken.de (A.F.); Annegret.Leuner@elblandkliniken.de (A.L.); Martin.Wolz@elblandkliniken.de (M.W.); 5 Chair of Business Informatics, esp. Systems Development, Faculty of Business and Economics, Technical University of Dresden, 01062 Dresden, Germany; carola.gisske@tu-dresden.de (C.G.); hannes.schlieter@tu-dresden.de (H.S.); 6 Center for Evidence-based Healthcare, Faculty of Medicine Carl Gustav Carus, Technische Universität Dresden, 01307 Dresden, Germany; claudia.helmert@uniklinikum-dresden.de (C.H.); Andreas.Knapp@uniklinikum-dresden.de (A.K.); Caroline.Lang@uniklinikum-dresden.de (C.L.); Gabriele.Mueller@uniklinikum-dresden.de (G.M.); 7 Vilans, National Centre of Expertise in Long Term Care, 3527 GV Utrecht, The Netherlands; M.Minkman@vilans.nl (M.M.N.M.); N.Zonneveld@vilans.nl (N.Z.); 8 Tilburg University/TIAS School for Business and Society, 5037 AB Tilburg, The Netherlands; 9 Department of Neurology, University Hospital Marburg, 35043 Marburg, Germany; munster@med.uni-marburg.de; 10 Department of Neurology, Klinik am Tharandter Wald Hetzdorf, Herzogswalder Straße 1, 09633 Halsbrücke, Germany; themann@reha-hetzdorf.de

**Keywords:** Parkinson disease, consensus, clinical pathway, integrated delivery of health care, patient care team, community networks, patient monitoring, telemedicine

## Abstract

As integrated care is recognized as crucial to meet the challenges of chronic conditions such as Parkinson’s disease (PD), integrated care networks have emerged internationally and throughout Germany. One of these networks is the Parkinson Network Eastern Saxony (PANOS). PANOS aims to deliver timely and equal care to PD patients with a collaborative intersectoral structured care pathway. Additional components encompass personalized case management, an electronic health record, and communicative and educative measures. To reach an intersectoral consensus of the future collaboration in PANOS, a structured consensus process was conducted in three sequential workshops. Community-based physicians, PD specialists, therapists, scientists and representatives of regulatory authorities and statutory health insurances were asked to rate core pathway-elements and supporting technological, personal and communicative measures. For the majority of core elements/planned measures, a consensus was reached, defined as an agreement by >75% of participants. Additionally, six representatives from all partners involved in the network-design independently assessed PANOS based on the Development Model for Integrated Care (DMIC), a validated model addressing the comprehensiveness and maturity of integrated care concepts. The results show that PANOS is currently in an early maturation state but has the potential to comprehensively represent the DMIC if all planned activities are implemented successfully. Despite the favorable high level of consensus regarding the PANOS concept and despite its potential to become a balanced integrated care concept according to the DMIC, its full implementation remains a considerable challenge.

## 1. Introduction

There is an increasing awareness of the importance of integrated care concepts (ICCs) for vulnerable patient populations of elderly patients with chronic diseases [1]. Patients with Parkinson’s disease (PD) are a prime example of such a vulnerable patient population. The disease often has a decade-long disease course, along which patients experience increasing and evolving symptoms with varying responsiveness to available therapeutic options, often accompanied by the need for complex therapies such as deep brain stimulation (DBS) or continuous infusion therapies [2]. In addition, PD is the second most common neurodegenerative disease and patient numbers are expected to double within 25 years [3,4]. Due to this, there is an increasing number of national and international ICC initiatives for PD patients [5].

PD care in Germany involves a large number of different healthcare providers and is hindered by fragmentation, lack of communication and coordination [6]. Although defined as a standard of care, the access to specialized treatment is highly limited, aggravating imbalances in care access and hampering the efficient delivery of individualized, multiprofessional care [7].

Eastern Saxony is a German region with approximately 50% of its population of 1.9 million living in rural areas and only one major city with a university hospital (Dresden, 530,000 inhabitants) [8,9]. In addition, the region is in a demographic transformation process with the oldest population of all German regions (mean age 46.2 years) [9]. Depending on the district, up to 40% of PD patients do not have regular access to neurologists or PD specialists, and up to 56% of all PD patients admitted to Dresden University Hospital are emergency cases (own calculations based on hospital admission information and based on secondary health data from the biggest regional statutory health insurer, AOKPLUS). This has led to a situation where core objectives in PD care are not met, such as timely and equal access to PD specialists and neurologists or the avoidance of disease-related complications and emergency admissions.

**Figure 1:**
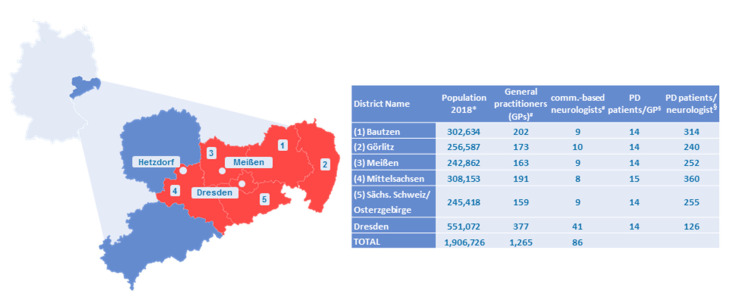
Characteristics of the intervention region Eastern Saxony. Germany is shown in light blue, the state Saxony in dark blue, and the intervention region Eastern Saxony in red. Within the intervention region, the three specialized hospital-affiliated outpatient centers are shown that will serve as the structural backbone of Parkinson Network Eastern Saxony (PANOS). The table on the right side gives population characteristics of the six districts within the intervention region. Eastern Saxony has a population of 1.9 million people, of which approximately 15,000 have Parkinson’s disease (PD). * General population numbers were taken from public statistical resources (https://www.statistik.sachsen.de/html/bevoelkerungsstand-einwohner.html). PD cases were calculated based on secondary health data from the biggest local statutory health insurer (AOKPLUS). Criteria were: International Classification of Diseases 10^th^ revision (ICD10) G20.x and prescription of dopaminergic medication as a validation criterion. The resulting prevalence of 786.69/100,000 matches the one of another recent German-wide epidemiologic study based on secondary health data [12,13] and was the basis for the ^§^ calculation of the number of patients per general practitioner (GP)/community-based neurologist. ^#^ Number of general practitioners (GPs) and number of neurologists was provided by the Association of SHI Physicians. Row “Total” gives the summed numbers for all six districts in the intervention region. Whereas the average number of PD patients per GP varies little between urban and rural districts (14 patients/GP), there was a huge variation in the average number of PD patients per neurologist between rural areas (up to 360 patients/neurologist in Mittelsachsen) and the city of Dresden (126 patients/neurologist).

Since 2017, a multiprofessional team consisting of community-based physicians, PD specialists, medical therapists, patients, experts for design and evaluation of ICCs, representatives of statutory health insurances (SHI) and local medical authorities has collaboratively developed a concept for a multimodal intersectoral ICC for PD patients, named Parkinson Netzwerk Ostsachsen (Parkinson Network Eastern Saxony; PANOS). To locate the region and its participating centers please refer to Figure 1.

Theoretical frameworks on ICCs and practical experiences stress the importance of a multidimensional strategy that addresses various aspects ranging from the definition of care delivery and quality standards, roles and tasks, to education and engagement measures for patients and healthcare providers [10,11]. The PANOS concept incorporates several of these multidimensional aspects and follows a sequential implementation strategy adopted to the regional healthcare context.

**Figure 2:**
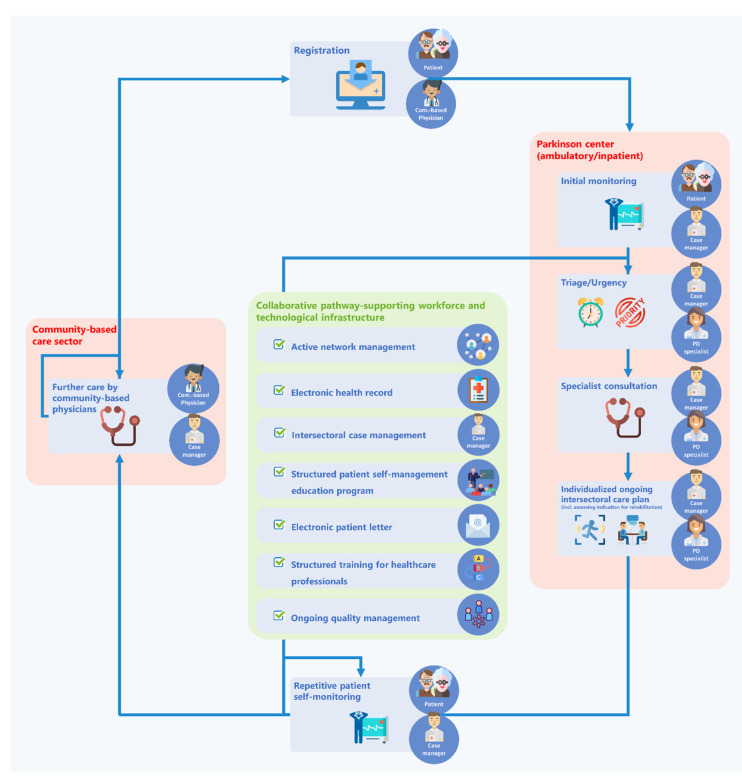
The PANOS care pathway with its supporting workforce and technological infrastructure.

It starts with the implementation of an intersectoral care pathway as the core and basis for standardized healthcare delivery (for details on the care pathway, see Table 1 and Figure 2). This care pathway is to organize and standardize the collaborative work of physicians at three specialized hospital-affiliated outpatient centers located in Dresden, Meißen and Hetzdorf and of community-based neurologists and general practitioners (GPs).

The implementation process will be accompanied by several supportive technical, professional and communicative measures (Table 1 and Figure 2).

In order to reduce implementation complexity, the ICC will be restricted to the region of Eastern Saxony during the first phase (Figure 1) and will focus on the coordination of care-providing physicians and case managers. The structured integration of further involved healthcare providers (e.g., physiotherapists) will be addressed after a successful implementation of the core elements described here.

PANOS will focus on the most vulnerable PD subpopulations, defined as:

1. patients in the transition phase from the early, uncomplicated disease stage (honeymoon) to advanced disease stages
2. patients in advanced disease stages
3. patients with an unsecured diagnosis and in need of specialist involvement in diagnosis finding, irrespective of the disease stage.

Since the consortium understands the acceptance of the concept as an indispensable prerequisite for a successful implementation, a modular-structured consensus process was organized, engaging representatives of all healthcare professionals and institutions that will participate in the implementation process.

The aim of the present paper is to describe the PANOS concept as the result of its sequential development process, including the structured consensus process. In addition, PANOS was evaluated according to a validated theoretical framework of integrated care (Development Model for Integrated Care; DMIC) [1]. The DMIC is an expert-consensus based holistic model of components relevant to the practical implementation of ICCs and allows for an assessment of both the comprehensiveness and the maturation stage of a single ICC.

## 2. Materials and Methods

### 2.1. Concept Development Overview

The PANOS integrated care concept was developed in a sequential interprofessional collaborative effort. This was realized by a series of six workshops (WS) in addition to numerous smaller workgroup meetings. The first three workshops were conducted as semistructured organized conferences to develop the core concept. The subsequent three workshops were executed as a structured consensus process in compliance with recommended scientific methodology [14].

### 2.2. Structured Consensus Process

Three iterative structured workshops meetings were conducted between January and June 2020, of which two were face-to-face and one was an online meeting due to the SARS-CoV-2 lockdown restrictions. Meetings were free-of-charge, and travel and other expenses were covered by project funds.

To ensure an equal level of information, participants were provided in advance with content material about PANOS through a condensed illustrative summary that was developed in the three former semistructured workshops. Preceding online surveys were carried out to retrieve participants’ broader perceptions and expectations on the status quo, challenges and potential benefits of an ICC.

Workshops were conducted following a three-stage procedure:

1. An initial input session in plenum provided participants with detailed information about the specific aspects/modules of the PANOS concept to be consented. A short discussion round followed to clarify potential misunderstandings.
2. To facilitate low-threshold, in-depth discussions, the panel was then split into three small moderated discussion rounds. The discussion rounds were guided by previously specified open questions, covered the content of the current workshop and also included the nonconsented aspects from the preceding workshop.
3. In the final session, Tele-Dialog votings (TED votings) were conducted to reach a consensus on relevant aspects of PANOS. Most TED questions were formulated prior to the workshops, but new aspects from the discussion rounds were included at the discretion of the organization committee.

The TED voting questions for the structured consensus purposely focused on those aspects of PANOS that were rated as the most relevant for the later intersectoral and multiprofessional collaborative work. Thus, these aspects were covered more intensively than other areas, and deemed less relevant to collaborative work (e.g., work organization within the centers).

Participants represented the following professions and/or institutions: physicians (20/18/13 for Workshop1/Workshop2/Workshop3), other healthcare providers (0/5/5), patient representatives (1/2/2), scientists (10/9/9), statutory health insurances (1/2/0), Association of SHI Physicians (1/1/0) and the Federal State Chamber of Physicians (0/2/0).

### 2.3. Data Analysis and Presentation

Based on the guidelines for structured consensus processes by the Association of the Scientific Medical Societies in Germany, a consensus was defined as an agreement of >75% [14]. The results were analyzed using descriptive statistics. All TED voting results are presented in the present paper. All items were assigned acronyms to match the respective consented item in the tables to its reference in the text (Table 2). Due to space restrictions, a selection of the total 165 online voting results was included, as selected by an independent three-level rating by the authors (for all online voting results, see Supplementary Table S1). Group discussion rounds were protocolled during the session, audio recorded and transcribed.

### 2.4. Application of the Developmental Model for Integrated Care (DMIC)

Six key representatives from all project partners actively involved in the conceptual design process did an independent online-rating of the PANOS according to the DMIC. The results were analyzed according to the established analysis protocol of the DMIC [1].

## 3. Results

### 3.1. Structured Consensus Process

The group size was comparable in workshop 1 and 2 (34 and 39 participants) and was smaller in workshop 3 (29 participants) due to the current SARS-CoV-2 situation and special event format.

A total of 228 items was presented to participants in the course of all three workshops, 165 thereof in preceding surveys and 62 in TED votings. For the domains of the PANOS concept covered, please refer to Table 2.

**Figure 3:**
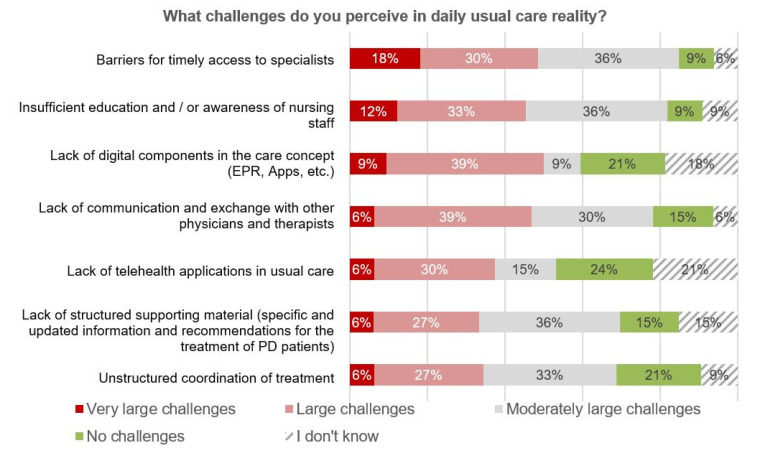
Survey results—perceived challenges in usual care reality (*n* = 33).

As part of the online votings, participants were asked to rate the challenges they perceive in daily usual care (Figure 3). A timely access to specialized care, as one primary goal of PANOS, was rated as the most relevant current barrier, followed by an insufficient education of healthcare professionals and by the lack of interprofessional cooperation and communication, associated with a lack of appropriate technical infrastructure for collaborative work. Most of these regionally recognized problem areas are in line with both patient- and expert-based perceptions of relevant areas of improvement in PD care throughout the world [15,16,17].

In the following sections, the workshop results are clustered according to the main domains of the PANOS concept (for an overview, see Table 1 and Figure 2). A short description is provided, followed by the related results from online surveys (if applicable) and consensus results.

### 3.2. Consensus about the Core Elements of the Standardized Integrated Care Pathway PANOS

1. Patient registration (REG)

In order to allow for a timely and equal access, easy-to understand, low-threshold clinical registration criteria were formulated under consideration of the international expert-based consensus processes for the definition of advanced PD [18] (Table 3; Table 4, REG2). However, these criteria were adopted to facilitate an easy understandability for both patients and GPs, and to be more sensitive to patients in the early transition phase from early to advanced PD. Since up to 40% of PD patients in the region of Eastern Saxony do not have access to a community-based neurologist, registration must also be possible for GPs (Table 4, REG5). Inscribing physicians must provide information about urgency. A professional registration will be carried out via the web-based electronic health record (EHR) (Table 4, REG1), but an additional paper-based registration and a patient self-assessment tool will be provided (Table 4, REG3, 4). Since the varying motivation of >1.300 potentially registering physicians in Eastern Saxony could present a considerable bottle-neck, self-registration may exceptionally be undertaken by patients themselves (Table 4, REG3, 4).

However, patient self-registration raised concerns and was only consented after more detailed explanations were provided. The major concern was that it could present an uncontrollable bias to physician-based eligibility selection. The following procedural steps enabled the consensus: the case manager reviews all (self-) registrations and always informs the treating community-based physician in case one of his/her patients chooses self-registration. In case of dissent about eligibility, indicated urgency, or self-registration, the case manager will try to achieve an interprofessional consensus (collaborative consensus principle of PANOS) (Table 4, REG6).

Registered patients are to be distributed between the three Parkinson centers according to their zip codes. Deviation from this principle might occur in case a strong imbalance in patient load develops between the three centers (Table 4, REG6).

2. Pre-consultation patient self-monitoring and baseline information collection (PCM)

Within PANOS, patients are to assume an active role in their own healthcare. In order to prepare patients for a more active role, a standardized patient education will be offered to promote self-management capacities (see below). One important self-management competence is self-monitoring [19]. Before getting their first consultation in a Parkinson center, patients will be asked to fill in a standardized self-monitoring package at home, containing validated self-assessment tools for motor and non-motor symptoms and for psychosocial health domains (Table 4, PCM1).

In addition, patients will be asked to contribute as much as possible to the gathering of their medical history as the baseline for the ongoing longitudinal care within PANOS. Patients will be supported by their individually assigned case manager and several iterations might take place, as long as the patient’s condition and the urgency as indicated by the inscribing physician permit this. The rational for this approach is to both involve the patient as an active partner and to allow for an efficient collection of standardized health-related information.

Self-monitoring packages will first be provided as machine-readable paper-based questionnaires in order to not exclude patients without sufficient digital competence. However, electronic patient self-monitoring (e.g., app-based), as well as sensor-based monitoring, are envisaged as the proximal expansion stages of PANOS. Returned information will be processed semiautomatically and integrated into the EHR.

3. Triage (TRI)

Based on the comprehensive information gathered by the patient-based pre-consultation monitoring, all consultations will be organized based on a triage system with the criteria of urgency (emergency, urgent or regular) and expected complexity and associated time need (Table 4, TRI1). Although a clear goal of PANOS is to promote outpatient care, inpatient hospital admissions can be initiated if required by the patient’s condition.

4. Structured specialist consultation

Consultations with PD specialists in Parkinson centers will be based on a standardized process with clearly defined responsibilities among the center staff in order to ensure efficient workflows. The consultation duration and agenda are determined by the preceding triage process and the information available due to the pre-consultation patient self-monitoring.

Consultations will be divided into a non-medical visit with the case manager personally assigned to an individual patient and a subsequent medical consultation with the PD specialist. The case manager complements missing monitoring information together with the patient and performs additional professional tasks as assigned. The specialist can then base his/her consultation on this prior work contributed by the patient and his/her case manager. The entire workflow and all required documentations will be reflected by the EHR.

Work organization during structured consultations within the Parkinson centers, albeit discussed in the workshops, was not a subject matter of the structured consensus because of the deliberate focus on intersections relevant to intersectoral collaborative work organization.

5. Individualized ongoing intersectoral care plan (ICP)
   At the end of each center consultation, specialists are to suggest an individualized ongoing care plan for the following 12 months to all other involved healthcare providers and the patient (Table 4, ICP1). As part of this care plan, the following aspects have to be determined:
  (a) Frequency, time frames and relative professional contribution (community-based physician vs. specialist) to future scheduled outpatient consultations within PANOS. Depending on the individual patient’s condition, all distributions are possible, ranging from 100% care provision by community-based physicians (for patients still in the early transition phase) to a 100% care provision by Parkinson centers (for patients with complex therapeutic needs or important complications)
  (b) Structured eligibility assessment for inpatient rehabilitation programs
  (c) Recommendations for frequency and therapeutic objectives of active therapies (physiotherapy, occupational or speech therapy)
  (d) Individualization of content or frequency of the predefined packages of the repetitive patient self-monitoring

This individualized ongoing care plan is understood to be an important instrument to allow for efficient resource allocation. The low-threshold clinical registration criteria imply that patients with important variations in therapeutic needs will be treated in PANOS. Without an element for individualized need-adjusted care intensity within the standards of the care pathway, an economic care delivery would be severely compromised.

Care plans as suggested by PD specialists will be shared via the EHR. In case of dissent, all involved healthcare providers can suggest changes until a mutual consensus is reached.

6. Repetitive patient self-monitoring (MON)

All patients registered in PANOS will be asked to complete quarterly standardized self-monitoring packages.

In addition to the general arguments given above for self-monitoring, the quarterly repetitions are to function as a safeguard for the early detection of relevant changes in patient conditions, independent of the individualized ongoing care plans. Different content volumes will be defined for every 3, 6, and 12 months. Both frequency and volume can be individualized to exceed the predefined minimal monitoring standard if a patient’s specific situation warrants this. The monitoring results are recorded in the EHR and can be accessed by all relevant EHR-users.

Having repetitive detailed information about a patient’s condition means a gain in responsibility to take timely action. In order to assure this and not to overcharge community-based physicians, the main responsibility will be taken by the staff of the Parkinson centers (Table 4, MON3).

Community-based physicians need explanations of the instruments and on the interpretation of results that will be displayed on the EHR (Table 4, MON2). The option for community-based physicians to adjust the monitoring packages was not consented (Table 4, MON1).

These results are in line with preceding split-group discussions. GPs especially expressed the concern of potentially being overcharged by more detailed insights into a patient’s condition as provided by the repetitive monitoring.

7. Structured consultation with community-based physicians (CBP)

For all patients who do not require regular specialist consultations, the individualized ongoing care plan might envisage all or the majority of ongoing care to be provided by the collaborating community-based physician (e.g., patients in the late stages of their honeymoon phase, or early stages of transition phase). In order to enable collaborative work, all healthcare providers will have full access to the EHR, and their tasks will be defined by a structured workflow integrated into the EHR. Since both the extent of standardized responsibilities and the related design of data visualization will have an impact on the willingness to become an active contributor to PANOS, both aspects received substantial coverage. Even if registered in PANOS, not all patients will have access to community-based neurologists, and therefore some GPs will become active long-term contributors in collaboration with the Parkinson centers. It is therefore of high importance to understand and meet the needs of a diverse group of potential active contributors.

**Figure 4:**
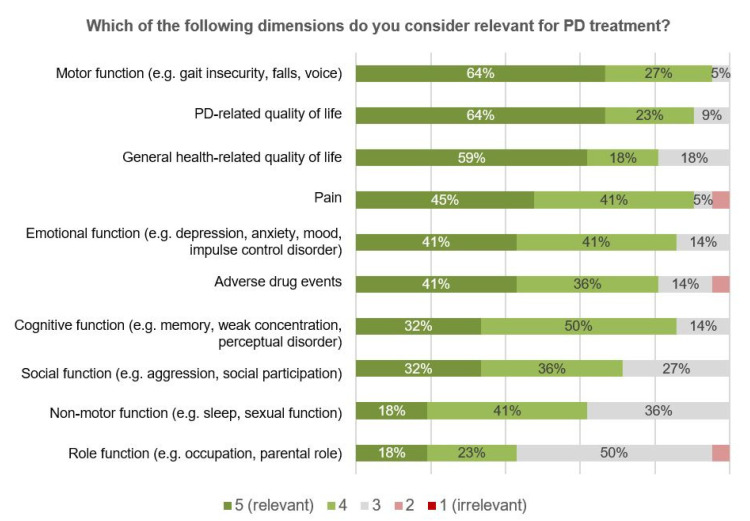
Survey results—relevant dimensions for PD treatment from physicians’ perspectives (*n* = 21).

As part of the online surveys, participants were asked to prioritize the monitoring of different PD-related symptoms, and of functional and psychosocial health-related domains (Figure 4). By rating motor symptoms as the most, and PD-related quality of life (QoL) as the second most, important aspect, participants recognized the importance of monitoring different levels of health in PD, ranging from symptoms, over functions to the overall impact on the patient’s QoL [20]. In general, a lower relevance was attributed to psychosocial health-related domains such as role or emotional functioning.

Regarding the work distribution between Parkinson centers and community-based physicians, the maintenance of the medication plan was consented to be a responsibility of community-based physicians (Table 4, CBP1), but not the conduction of standardized clinical tests, such as the MoCA (Table 4, CBP2). This was rather agreed to be a responsibility of the center-affiliated case managers (see below at intersectoral specialized case management).

8. Additional time requirements of community-based physicians

Additional time requirements for community-based physicians are to be expected due to participation in PANOS. In order to account for this, there will be a supplementary payment of EUR 25–35. Acceptable additional time requirements were discussed in the split-group discussion rounds, and averaged acceptable durations from the split-group discussions were included in subsequent TED votings. As it already became evident in the discussion rounds, there was a huge variability in the time spent on an individual PD patient’s community-based care, accompanied by a large variation in the acceptable additional time requirements. Therefore, no overall consensus could be achieved (Table 4, ATR1–7). However, under consideration of the supplementary payment, it was consented that each neurologist consultation could be extended by an additional 15 min (Table 4, ATR6), and that there could be up to one additional quarterly consultation (Table 4, ATR7).

### 3.3. Supportive Personal, Technical and Communicative Measurements

1. Electronic health record (EHR)

An EHR tailored to the specific use case of the PANOS is regarded as a crucial basis for efficient and truly collaborative structured intersectoral care. The EHR will be a web-based application that visualizes not only all relevant clinical information (e.g., medical reports, diagnostic test results, results of the repetitive patient self-monitoring), but will also define workflows and associated tasks as part of the standardized care pathway. Where relevant and feasible, the interoperability to other EHRs is planned (e.g., the online communication and billing system by the German Association of SHI physicians KV-SafeNet). Both the definition of acceptable work packages and a high-quality user interface (UI) will have an important impact on efficiency, quality of care and on the professional motivation to become a reliable contributor.

**Figure 5:**
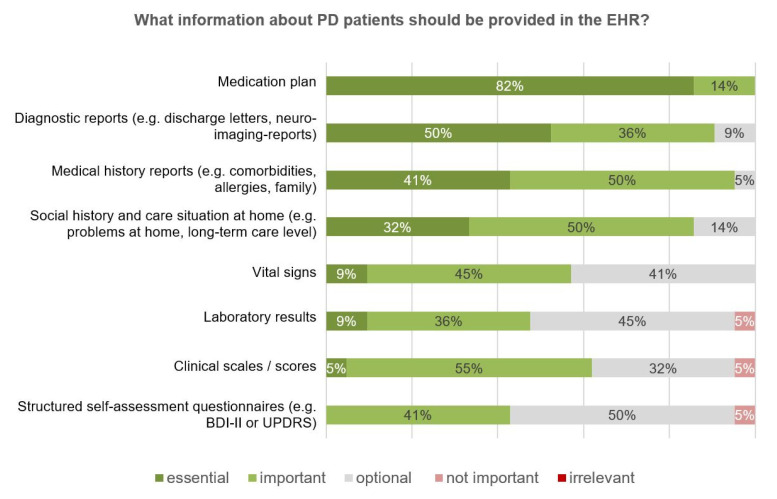
Survey results—information about PANOS patients provided in the electronic health record (EHR) (*n* = 21).

The relevance of different medical information to be provided in the EHR was assessed, with the medication plan ranking the highest (Figure 5). Despite the overall acceptance of the concept of repetitive monitoring, the provision of the respective results had the lowest priority of all the medical information assessed.

All contributing community-based physicians will actively use the EHR as the basis for standardized care and documentation (Table 5, EHR 1–4).

Largely corresponding to those health-related dimensions where the availability of information was valued as the most meaningful (Figure 4), community-based physicians were also expected to actively contribute to their collection (Table 5, EHR 5–14). In line with this, a contribution of community-based physicians was consented in functional domains (Table 5, EHR 5–9), in contrast to the contribution in psychosocial domains (Table 5, EHR 11, 14). An exception was the information about QoL, valued as the second highest relevance (Figure 4), but where a contribution to information collection by community-based physicians was not consented (Table 5, EHR 12, 13). The assessment of QoL was rather regarded as a responsibility of case managers.

Repetitive quantitative data, e.g., scores from self-assessments tools, should be visualized as a color-coded heatmap (Table 5, EHR 16) and should be customizable, e.g., by (de-) selecting scores (Table 5, EHR 15).

There was a clear preference for closed selection fields over semi- or unstructured open text fields for documentation (Table 5, EHR 21, 19, 20).

2. Intersectoral specialized case management (ICM)
   Case managers are pivotal for PANOS as they hold the major linkage between the outpatient sector and Parkinson centers. The team of case managers will represent the personal backbone of PANOS with an array of core responsibilities (Table 6, ICM 1–7, Table 4, MON 3):
  - To be the long-term one-spot contact person for individually assigned patients and for all of their healthcare providers;
  - To perform patient home consultations and home-based social assessments;
  - To assure the structured availability of required clinical information with a special focus on the repetitive patient self-monitoring;
  - To assure timely reactions of Parkinson centers in case of relevant changes in the monitoring results;
  - To plan and execute measurements for the active network management;
  - To execute the patient education program after adequate training (train-the-trainer principle);
  - To execute continuous quality control according to the quality management concept (see below).

Case managers will be prepared in a modular training program before taking up the above-mentioned diverse tasks. In order to support community-based physicians in their work in PANOS, services can be requested from case management (Table 6, ICM 6).

3. Active network management (ANM)

Mobilization and motivation of community-based physicians is not only an essential prerequisite to assure an equal and timely access of eligible patients, but also for the collaborative concept of an intersectoral care provision partnership.

This will be addressed by several strategies including: the structured acquisition of participants due to personal on-site consultations; releasing information updates about the network status; organization of educational symposiums; project discussion groups; topic-specific workgroups; stakeholder meetings and regional quality circles (Table 7, ANM 1, 5, 6). No consensus could be achieved on an adequate frequency of plenary meetings.

4. Structured patient education program according to self-management concept (EDU)

Structured patient education is deemed as an essential core measure within PANOS. It not only gives PD patients self-management education the highest priority when asked about expectations in the context of ICCs [15,17], but also deems expert panels self-management education measures as indispensable for healthcare delivery to chronic long-term conditions [21].

This especially holds true for concepts such as PANOS, where patients are assigned an active role in their own care. The more empowered patients are to do this, the better the chance that they can become a meaningful active contributor to their own care [22].

The self-management concept implies that PD-related knowledge is only one of several skills a patient has to master in order to induce and maintain health-promoting self-management behaviors. Developing beliefs regarding a sufficient self-efficacy as a core mediator for self-management behaviors, as well as additional skills such as action planning or adequate resource utilization, are indispensable for this [23].

Generally, a multi-modular, small-group, in-class setting is used for implementation. Informal caregivers are mostly included, since most patients rely on them in order to perform self-management behaviors successfully [24].

In Sweden, a sustainable nationwide implementation of a structured program for PD patients according to the self-management concept has been achieved in the recent years, including some evidence for its efficacy [25,26]. In order to allow for a timely implementation within PANOS, the concept of the Swedish National Parkinson School will be adopted together with the Swedish program initiators.

**Figure 6:**
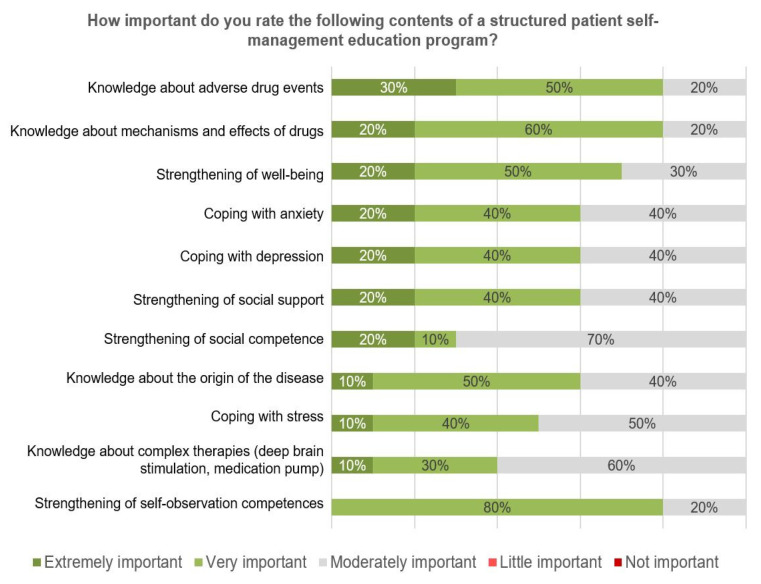
Survey results—importance of the contents of a structured patient education program (*n* = 10).

Participants were asked to rate the importance of potential curriculum topics. The highest relevance was given to a knowledge domain (adverse drug events), followed by self-management behaviors such as coping with emotional impact (Figure 6).

Both the general concept of a structured program according to the self-management concept and several knowledge domains were consented (Table 8, EDU 1–7).

Patients and caregivers will be provided with patient letters in lay language about clinical and treatment statuses and the care plan. Patient letters will be created automatically based on data recorded in the EHR using preset text modules, tested for patient-orientated comprehensibility.

5. Structured professional education curriculum

Both community-based neurologists and GPs are an integral part of PANOS. To enable participating physicians to perform their tasks in patient selection and in participating in ongoing care, a structured education curriculum for all professional care providers will be established. The physician education curriculum is to focus on both the timely recognition of patients in the transition phase and in need of specialized care, as well as on the knowledge and skills needed to become a productive active long-term collaborator. Given the special importance of GPs in care provision to PD patients in Eastern Saxony, an education module is planned to specifically target the needs of GPs (“PD for GPs”).

### 3.4. Assessment of PANOS according to the Developmental Model for Integrated Care (DMIC)

**Figure 7:**
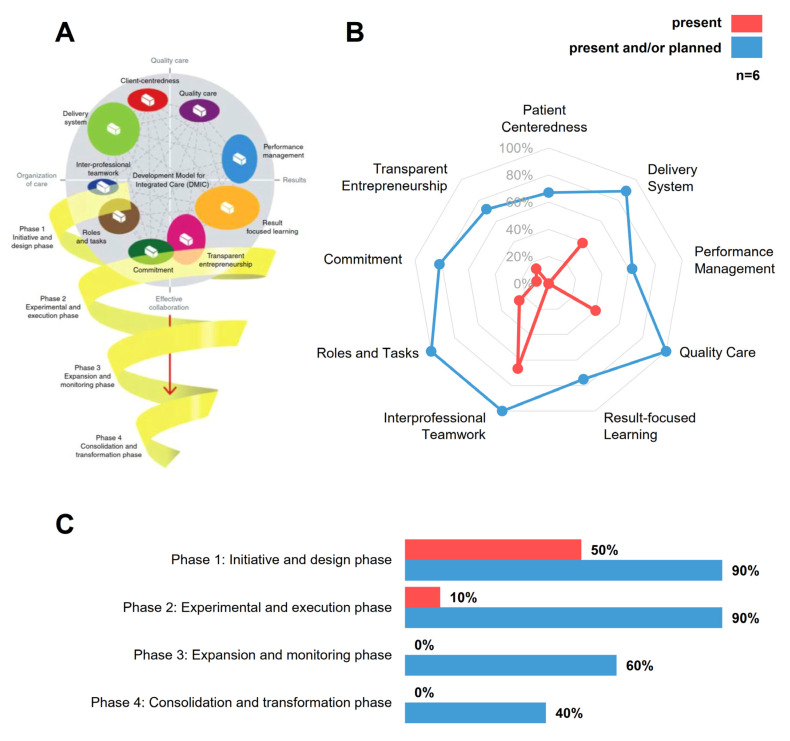
Assessment of PANOS according to the Development Model for Integrated Care (DMIC) framework (**A**): Illustrative summary of the Developmental Model of Integrated Care (DMIC). A total of 89 components relevant to ICC implementation are grouped into 9 clusters and located on a cluster map with 4 axes (Organization of care, Quality care, Results and Effective collaboration). The model also considers 4 developmental stages ranging from phase 1—initiative and design phase, to phase 4—consolidation and transformation phase. (**B**): Current assessment of PANOS by 6 involved key representatives according to which elements already have been achieved (rated as “present”, shown in red) and how PANOS would represent the model if planned activities are implemented (rated as “planned or present”, shown in blue). (**C**): PANOS is still in an early maturation state. Achieved elements belong to phase 1 and 2; the percentage of relevant elements already planned but not yet achieved is much higher for phases 1 and 2 (90% each) than for phase 3 (60%) and phase 4 (40%). Reproduced with permission from Mirella Minkman, Journal of Integrated Care; published by Emerald Publishing Pty Limited, 2016.

The DMIC is based on an expert-based Delphi-consensus about 89 components relevant to the practical implementation of ICCs [1]. These components are grouped into nine clusters and located on a cluster map with four axes (organization of care, quality care, results and effective collaboration) (Figure 7A). The model also considers four developmental stages ranging from phase 1—the initiative and design phase, to phase 4—the consolidation and transformation phase. It was validated with 84 different ICCs for a variety of diseases and settings, and it has already been applied in transcultural contexts (Netherlands, Canada). Taken together, the DMIC allows a structured assessment of ICCs regarding their representation of the multidimensional clusters and their developmental stage.

Six key representatives from project partners actively involved in the conceptualization of the care pathway independently assessed PANOS according to the DMIC theoretical framework. The assessments showed that PANOS is still at an early maturation state, with only low percentages of elements being fully implemented (Figure 7B, rated as “present”, shown in red). On average, the respondents evaluated the network with the highest scores of present elements on the clusters “Interprofessional teamwork” (67%), “Quality Care” (40%) and “Delivery System” (39%). The scores on the clusters “Patient-centeredness”, “Performance Management” and “Result-focused learning” are all 0%. However, if all “planned” and “present” activities are considered (Figure 7B), rated as “planned or present”, shown in blue, the PANOS concept represents the nine clusters in a balanced manner and to a high degree ranging from 63% (performance management) to 100% (roles and tasks, interprofessional teamwork and quality care). Ratings of the six representatives were more homogenous about planned elements than about the elements already present (not shown), indicating a stronger consensus regarding what is planned than about what has already been achieved. The conceptualization and implementation strategy of PANOS appears to be in compliance with the four sequential maturation stages of the DMIC. In total, 50% of elements of phase 1 (initiative and design phase) have already been implemented, followed by 10% of phase 2 (experimental and execution phase), and 0% for the more mature phases 3 and 4 (Figure 7C). Up to 90% of elements in phase 1 and 2 are already planned, followed by 60% and 40% for elements in phases 3 and 4, respectively. Thus, both the current maturation state (“present” elements) and the body of elements foreseen but yet to be executed (“planned” elements) appear to be well aligned with the four hierarchical developmental stages of the DMIC.

## 4. Discussion

### 4.1. Results of the Structured Consensus Process

All critical core elements and principles of the PANOS care pathway could be consented by a structured consensus process in compliance with the guidelines of the Network of Scientific Medical Societies in Germany (AWMF) [14]. Namely, the registration process and inclusion criteria, the concept of (repetitive) patient self-monitoring as an integral part, the triage concept and the concept of an individualized ongoing care plan with a variable distribution between community-based care and specialist care clearly met the consensus criteria of >75% agreement. Non-consented aspects, such as certain responsibilities for information collection in some relevant health-related domains, are not crucial to the core concept and can be addressed during the future in-depth planning process. The consensus process thus confirmed high levels of support for the core principles and provided valuable information for future planning and decisions during the implementation process.

### 4.2. PANOS in Comparison to Other PD-Specific Integrated Care Concepts

There is a growing number of ICCs for PD patients worldwide and in Germany [5]. This is prompted by the high relevance attributed to ICCs in assuring an adequate healthcare delivery for vulnerable patient populations [1] and by the urgency to find adequate solutions meeting the complex therapeutic needs of a rising number of PD patients [4,27].

However, there is no standardized agreement on the most relevant elements for an ICC for PD patients or for a sequential implementation strategy. A recent expert-based consensus sums 30 recommendations about crucial elements for PD ICCs, but this still leaves it open to regional initiatives to prioritize relevant elements [16]. In addition, all ICCs have to consider the regional context and region-specific needs and expectations of both patients and implementation partners [15,28]. Despite universal recommendations to ICC implementation, there will always be the challenge to interpret their relevance for the specific regional context.

Existing PD networks differ both in their maturity and in their implementation strategy. ParkinsonNet in the Netherlands played a pioneering role in implementing a community-based multidisciplinary network in 2004 [29]. The network focuses on the empowerment of patients and healthcare professionals through education, training procedures and evidence-based practice guidelines for almost all care disciplines. It includes a number of IT solutions to facilitate coordination among healthcare professionals and patients. There is evidence for the effectiveness and cost-effectiveness of some areas of activity, and for strengthened multidisciplinary collaboration [30,31]. Probably the most important advantage is the generalizability of the approach, which now has reached nationwide coverage in the Netherlands with 70 regional networks [32].

In the last couple of years, several PD networks were established in Germany. In 2018, Parkinsonnetzwerk Münsterland+ (PNM+) was established in Münsterland, a rural area in the north-west of Germany, based on a regionally modified concept similar to ParkinsonNet. The network involves inpatient care PD specialists, community-based physicians, and non-medical healthcare providers (e.g., different therapists) and focuses on collaborative network activities, as well as on the provision of comprehensive and easy-to apply care standards for all providers.

In Cologne, an ICC has been implemented based on regular PD specialist and PD nurse consultations to community-based neurologist practices. The network also offers patient education and video therapy. Another example is the Parkinson Netzwerk Allianz Marburg (PANAMA) (mid-west of Germany), that encompasses an array of initiatives, ranging from professional to patient-orientated education, specialized consultations in regional partnering hospitals and community-based practices, a modular intervention to strengthen patient’s emotional awareness to scientific projects on telehealth innovations.

In spite of similarities in some components, such as communicative network-promoting measures or professional and patient education, PANOS differs substantially. The most prominent difference is its focus on structured standardized care provision on the basis of an intersectoral care pathway and its inclusion of three hospital-affiliated outpatient specialist centers. This requires a substantially more technical and personal infrastructure than for the less formal approaches of the other above-mentioned ICCs.

### 4.3. PANOS Evaluated by the DMIC and Comparison to Patient and Expert-Based Recommendations

Key members from six different partners involved in the design, implementation and evaluation stages assessed the overall concept of PANOS (planned and present elements) to be a balanced concept according to the DMIC, incorporating components of all the four main axes of the model. However, only a few elements have been implemented (present elements). This implies that an array of design and implementation processes should be carried out simultaneously and that they increase the implementation complexity. Even if PANOS currently has a low maturation state (achieved 50% phase 1 elements, and 10% of phase 2), its sequential implementation concept is well aligned with the developmental stages with most of the elements planned in phases 1 and 2 (90% each), 60% for phase 3 and 40% for phase 4, respectively.

The DMIC provides practically applicable information for the future implementation process: it shows what level of planning still needs to be done for the future phases 3 and 4, and it provides information on which clusters need attention from the project team, e.g., it could be worthwhile to put a bigger emphasis on clusters where there is little consensus on the implementation status already achieved.

From a patient’s perspective, PANOS strives to address core requirements: according to a Dutch study carried out in compliance with the voice of customer (VoC) approach [17], the following are the most important patient requirements: (1) desire for self-management, (2) better collaboration between healthcare providers, (3) more time for discussing the future, (4) one healthcare provider who can act as a personal case manager, (5) more knowledge of the disease, (6) more support from my pharmacist, (7) increased focus on the needs of my spouse, (8) more contact with other patients, (9) more provision of information and (10) less fragmentation of healthcare. PANOS addresses aspects 1, 3, 5, 8 and 9 by incorporating a structured patient education program according to the self-management concept, aspects 2 and 10 by the establishment of a structured care pathway and aspect 4 by the provision of such a personal case manager.

In the light of a recent expert-based consensus on the recommended components of PD ICCs, PANOS considers several of the 30 recommended components [16]: follow-up consultations should be scheduled according to individual patients’ needs, a first point of contact should be provided, efficient interprofessional communication should be facilitated, support for self-management should be provided, and there should be a central care coordinator. In addition, PANOS also tries to contribute to digital innovations by developing a disease-specific EHR for intersectoral care.

Taking the consistency of the PANOS concept with patients’ needs, expert consensus-based recommendations and with the clusters of the DMIC, it could be postulated that core elements of the concept could also be valuable in other regional contexts with similar healthcare challenges as described for Eastern Saxony. However, careful adaptations to the specific implementation context will always be of outstanding importance, no matter which existing ICC might be considered as a starting point.

### 4.4. Implementation Risks and Perspectives

The need to alter existing healthcare structures in order to deliver care by an alternative (integrated) care concept implies significant risks to a successful implementation. A clear risk to the PANOS concept is that the implementation of a structured care pathway, albeit met by a high level of interprofessional acceptance, requires important supportive infrastructure, above all by a custom-made EHR. This is accompanied by the requirement to simultaneously organize workflows and to define roles and tasks under the appropriate consideration of the working reality of the healthcare providers needed as contributors. Thus, compared to other ICCs, the PANOS concept entrails are high level of implementation complexity. This is also reflected by the DMIC model that indicated a lot of relevant components are being worked on, but all at the same developmental stage.

Because of this, measurements have been undertaken to limit complexity, e.g., PANOS abstains in the first implementation phase from the structured integration of all relevant healthcare providers (e.g., physiotherapists), and the repetitive patient self-monitoring is carried out in a conventional paper-and-pencil format and not digitally. The structured consensus process represents an effort to limit risks by the establishment of a collaborative intersectoral working environment and by assessing the needs of all potential contributors adequately. Because of the comparatively high implementation complexity, an ongoing formal evaluation and measure for iterative procedural and technical adaptations will be important to assure an improved fit of both the PANOS concept and its technical basis to the actual needs and expectations.

Once the care pathway with specialists, community-based physicians and case managers as stakeholders of the first phase has been successfully implemented, an important perspective will be to integrate other relevant healthcare providers, such as physiotherapists or occupational therapists. The integration of repetitive patient self-monitoring with an associated medical data management strategy and the establishment of a disease-specific EHR represents an ideal basis for the implementation of digital patient monitoring and patient–physician interaction strategies. However, even if the integration of sensor-based monitoring and app-based interactions is already envisaged, this can only be successfully realized if the core concept as described is functional.

Another important challenge will be to assure sustainable financing beyond the current project phase. This will be both dependent on the illustration of the medical effectiveness in the accompanying summative evaluation (primary endpoint: health-related QoL) and on an analysis of the economic impact of the concept. Studies on the economic effects of ParkinsonNet in the Netherlands illustrate that ICCs for PD patients can be cost-efficient [30,32,33]. The cost efficiency will depend on changes in healthcare utilization behaviors (e.g., by lowering the number of unplanned unstructured emergency admissions), on the reduction in disease-related complications (e.g., falls and fractures), and on the dimension related to the extra costs associated with the PANOS concept. However, considering the substantial differences between PANOS and the other ICCs described above, cost efficiency cannot be extrapolated and has to be illustrated for the specific concept.

### 4.5. Limitations of the Structured Consensus Process

Even though participants of the workshops came from various targeted professions and/or institutions relevant to the implementation of PANOS, the workshop participants cannot be considered to be representative for the full spectrum of healthcare providers, especially community-based physicians. The actual numbers of participants were well suited for the chosen concept of a structured consensus process and were within in the limits recommended. However, up to 1300 community-based physicians would have been eligible for workshop participation, but the highest number of participants from this group was only 20. Thus, a likely (and in our eyes unavoidable) recruitment bias in favor of those ready for healthcare innovations has to be accounted for when interpreting the results of the consensus process. The PANOS concept should not be expected to meet the same high level of acceptance in the real-world implementation scenario now to follow. It will be an important challenge to the project team to consider this adequately, in spite of encouraging the high support of the contributors to the current structure of the consensus.

## Figures and Tables

**Table 1:** Care pathway elements and supportive measures.

No.	Name	Description
	Care Pathway Components	
1	Registration	To assure timely and equal access for all eligible patients, registration has to be low-threshold with easy-to understand clinical registration criteria and registration rights to all community-based neurologists, GPs and patients themselves.
2	Pre-consultation patient self-monitoring	Prior to a consultation at one of the specialized centers (“Parkinson center”), patients will receive standardized self-monitoring packages to ensure availability of relevant patient information.
3	Triage	Based on the pre-consultation self-monitoring, patients will be triaged according to the criteria of urgency and expected therapeutic complexity.
4	Specialist consultation	Tasks and responsibilities will be clearly defined and assigned among center staff to allow physicians to focus on the medical core aspects.
5	Individualized ongoing intersectoral care plan	Following a consultation, specialists are to plan the relative contribution of the Parkinson center and the treating community-based physician on an individual patient-to-patient basis.
6	Repetitive patient self-monitoring	All patients will receive self-assessment monitoring packages at quarterly intervals to allow for timely detection of changes in condition.
7	Consultation with community-based physician	As planned by the individualized ongoing intersectoral care plan, patients are seen by their community-based physician whose responsibilities are defined by the care pathway. If indicated, the physician can prompt changes in the treatment plan, e.g., by asking for an intensified contribution of the Parkinson center.
	**Supportive personal, technical and communicative measures**	
1	Electronic health record (EHR)	All patient-related information will be recorded in a collaborative electronic patient management/documentation platform that all involved healthcare providers have access to.
2	Intersectoral specialized case management	A team of case managers who specialize in PD care will be the personal backbone of the Parkinson Network Eastern Saxony (PANOS). They will serve as an individual patient’s care coordinator and as the first contact person to the patient and all involved healthcare providers. In PANOS, they will be additionally responsible for network management activities, carrying out the structured patient school and support physicians in Parkinson centers and private practices.
3	Active network management	Ongoing mobilizing initiatives to promote the motivation of community-based physicians to become an active collaborator.
4	Structured patient school according to self-management concept	Modular group-based patient education program to promote self-management competences.
5	Electronic patient letter	A patient-orientated version of medical documents, automatically generated based on the information available in the EHR.
6	Structured professional continuous education curriculum	An education curriculum will be developed addressing the specific education needs of both neurologists and GPs.
7	Ongoing quality management	Ongoing quality management to assure adherence to process standards and an equal quality of care provided across the entire PANOS network.

**Table 2:** Workshop contents allocated to their respective integrated care concept (ICC) domains.

Acronym *	Domain	Total No. of Questions	Consented (>75%)
Core Elements of the Standardized Integrated Care Pathway			
REG	Registration	6	5 (83%)
PCM	Pre-consultation self-monitoring and baseline information collection	1	1 (100%)
TRI	Triage	1	1 (100%)
ICP	Individualized ongoing intersectoral care plan	1	1 (100%)
MON	Repetitive patient self-monitoring	3	2 (67%)
CBP	Structured consultation with community-based physicians	2	1 (50%)
ATR	Additional time requirements to community-based physicians	7	5 (71%)
**Supportive personal, technical and communicative measurements**			
EHR	Electronic health record	21	14 (67%)
ICM	Intersectoral specialized case management	7	7 (100%)
ANM	Active network management	6	3 (50%)
EDU	Structured patient education program according to self-management concept	7	7 (100%)
**Total**		62	47 (76%)

* Acronyms will be used for reference purposes throughout the manuscript. REG: Registration; PCM: Pre-consultation self-monitoring and baseline information collection; TRI: Triage; ICP: Individualized ongoing intersectoral care plan; MON: Repetitive patient self-monitoring; CBP: Structured consultation with community-based physicians; ATR: Additional time requirements to community-based physicians; EHR: Electronic health record; ICM: Intersectoral specialized case management; ANM: Active network management; EDU: Structured patient education program according to self-management concept.

**Table 3:** Inclusion criteria to PANOS.

Clinical Registration Criteria for PANOS (at Least One of the Following)	
Motor Symptoms	Slow movements >2 h per day Distressing involuntary movements > 1 h per day Short-acting L-Dopa ≥ 4 doses per day Tremor without medication response Falls
Nonmotor Symptoms	Hallucinations, Psychosis Behavioral disorders, impulse control disorder Severe daytime sleepiness Persistent depression, anxiety

**Table 4:** Results of Tele-Dialog (TED) voting—core elements of the PANOS patient pathway.

Acronym *	Issue		Agreement	Consensus?
Core element 1: Registration				
REG1	Technical requirements	Are you technically able to register a patient in your practice via a web-based platform?	83%	Yes
REG2	Inclusion criteria	Do you generally support the inclusion criteria (diagnostic criteria) for people with Parkinson’s disease?	95%	Yes
REG3	Patient self-registration	Would you agree with self-registration by patients?	75%	No
REG4		Do you agree with the self-registration process for patients as presented?	83%	Yes
REG5	Patient registration by physicians	Do you agree with the registration process for participating physicians as presented?	96%	Yes
REG6	Patient allocation	Do you agree with the patient distribution concept between Parkinson centers and community-based physicians as presented?	94%	Yes
**Core element 2: Pre-consultation monitoring**				
PCM1	Pre-consultation monitoring process	Do you agree with the process of collecting all preliminary information for the initial registration as presented?	95%	Yes
**Core element 3: Triage**				
TRI1	Triage	Do you agree with the process of triage as presented?	100%	Yes
**Core element 5: Subsequent intersectoral care plan**				
ICP1	Care plan	Do you agree with the subsequent intersectoral care plan as presented as the basis for scheduling consultations within the collaborative PANOS network?	94%	Yes
**Core element 6: Repetitive patient self-monitoring**				
MON1	Monitoring contents	As a community-based physician, do you want to be able to change the repetitive monitoring?	58%	No
MON2		For your work as a community-based physician, do you need explanations for the interpretation of monitoring test results, e.g., in the form of an explanatory field?	87%	Yes
MON3	Responsibility of case managers	Do you agree that case managers, in cooperation with specialists at the Parkinson centers, have the main responsibility for an adequate patient-centered response to the results of the repetitive monitoring?	89%	Yes
**Core element 7: Consultation with community-based physician**				
CBP1	Responsibilities of neurologists	One of the responsibilities of community-based neurologists in PANOS is the maintenance of the patient’s medication plan.	86%	Yes
CBP2		One of the responsibilities of community-based neurologists in PANOS are cognitive tests.	75%	No
**Time investment in care within PANOS**				
ATR1	Time with GP	A PANOS consultation with a general practitioner may last 20 min.	100%	Yes
ATR2	Time with neurologist	A PANOS consultation with a community-based neurologist may last 30 min.	56%	No
ATR3	Time for consultation	Recording medical history should last 10 min.	71%	No
ATR4		The physical examination should last 5 min within the framework of PANOS.	86%	Yes
ATR5		The subsequent consultation should last 7 min.	79%	Yes
ATR6	Reimbursement	Considering an additional reimbursement, the consultation with the patient concerned may last an additional 15 min.	86%	Yes
ATR7		On average, physicians would see a PANOS-patient 1 additional time for an additional payment of about 35 €.	86%	Yes

Consensus was achieved if there was >75% approval. Green font and background color: Consensus achieved; Red font and background color: Consensus not achieved. REG: Registration; * PCM: Pre-consultation self-monitoring and baseline information collection; TRI: Triage; ICP: Individualized ongoing intersectoral care plan; MON: Repetitive patient self-monitoring; CBP: Structured consultation with community-based physicians; ATR: Additional time requirements to community-based physicians.

**Table 5:** Results of TED voting—electronic health record.

Electronic Health Record (EHR)				
Acronym *	Issue	Question	Agreement	Consensus?
EHR1	Access and usage of EHR among neurologists	Should community-based neurologists be prepared to actively work with the EHR in PANOS?	100%	Yes
EHR2		Should community-based neurologists have access to the EHR in PANOS?	100%	Yes
EHR3	Access and usage of EHR among GPs	Should general practitioners be prepared to actively work with the EHR in PANOS?	95%	Yes
EHR4		Should general practitioners have access to the EHR in PANOS?	95%	Yes
	Dimensions documented by community-based physicians	(If applicable depending on the health status of the person concerned and at different time intervals)- Within PANOS, I would like to document the dimension…		
EHR5		… “adverse drug reaction”	100%	Yes
EHR6		… “cognitive function” (e.g., memory)	93%	Yes
EHR7		… “physical function” (motor abilities)	86%	Yes
EHR8		… “physical function” (nonmotor abilities)	86%	Yes
EHR9		… “emotional function” (e.g., depression)	86%	Yes
EHR10		…”pain”	86%	Yes
EHR11		… “social function” (e.g., aggression)	71%	No
EHR12		… “PD-related quality of life”	71%	No
EHR13		… “general health-related quality of life”	64%	No
EHR14		… “role function” (e.g., professional activity)	57%	No
EHR15	Display of information	Should the visualization of repetitive monitoring results be customizable for you, e.g., by selecting or deselecting individual scores?	100%	Yes
EHR16		Do you agree with the visualization concept of repetitive data in the style of a color-coded heatmap?	94%	Yes
EHR17		Do you, as a community-based physician, only want to have a basic set of the results of the repetitive monitoring displayed in the EHR (if there is the additional option to retrieve detailed information)?	50%	No
EHR18vor 17		Do you, as a community-based physician, want to have all results of the repetitive monitoring displayed in the EHR?	81%	Yes
EHR19		For the medical history form I need several structured fields in subcategories in the EHR—each with free text only.	14%	No
EHR20		For the medical history form I only need a free text field.	14%	No
EHR21		For taking the medical history I would like to have mainly closed selection fields (and the option to use an additional free text field).	93%	Yes

Consensus was achieved if there was >75% approval. Green font and background color: Consensus achieved; Red font and background color: Consensus not achieved. * EHR: Electronic health record.

**Table 6:** Results of TED voting– intersectoral specialized case management.

Intersectoral specialized case management (CM)				
Acronym *	Issue	Question	Agreement	Consensus?
ICM1	Accessibility of CM	The case management should be easy to reach, also in writing, by the physicians in PANOS.	100%	Yes
ICM2	Tasks and responsibilities of CM	The presented case management tasks (and the additional social-advisory responsibilities, consultation management) are important aids for the community-based physicians.	100%	Yes
ICM3		Do you agree with the presented case management tasks in PANOS? **	100%	Yes
ICM4		Do you agree that the case management acts as a central contact person in PANOS?	100%	Yes
ICM5		Should cognitive tests be conducted by case managers?	96%	Yes
ICM6	Contents of service requests	Do you agree with the contents of service requests by community-based physicians to the case management of PANOS? ***	100%	Yes
ICM7	Contents of socio-medical assessment	Do you agree with the contents of the socio-medical assessment as presented? ****	100%	Yes

A consensus was achieved if there was > 75% approval. Green font and background color: Consensus achieved; Red font and background color: Consensus not achieved. * ICM: Intersectoral specialized case management. ** Case management tasks are: assessment/monitoring, support of social counselling, consultation management, patient training, implementation of home consultations, support of community-based physicians, quality assurance and data protection. *** Service requests include: support of the assessment of patients, preparation of medical consultations, processing of new requests, platform data maintenance, ensuring repetitive monitoring, reactions to results, carrying out tests that can be delegated by doctors, such as the Barthel index, among others. **** Contents of the social assessment are: Schooling and vocational training, Family relationships, Living environment, Social services, Mobility, Cognition and behavior, Self-care options (in the sense of activities of daily life), Therapy and treatment (e.g., speech therapy, occupational therapy, physiotherapy, medical consultations, etc.), Housekeeping, Financial and official business, Precautionary power of attorney, patient’s living will and care, Medical history, Documentation of the burden of care for caregivers.

**Table 7:** Results of TED voting—network management.

Active Network Management (ANM)				
Acronym *	Issue	Question	Agreement	Consensus?
ANM1	Relevance of plenary meetings	Do you agree with the idea of conducting regular plenary meetings with all PANOS partners at which PANOS-relevant topics are presented and jointly developed?	92%	Yes
	Time intervals of plenary meetings	Concerning the time intervals of possible plenary meetings: Do you agree with the option of holding plenary meetings with all PANOS partners at which PANOS-relevant topics are presented and jointly developed every…		
ANM2		…3 months?	0%	No
ANM3		…6 months?	67%	No
ANM4		…12 months?	44%	No
ANM5	Update about PANOS	Would you like to receive a regular newsletter informing about developments in PANOS?	92%	Yes
ANM6	PANOS quality circles	Can you imagine taking part in your own regional PANOS quality circle, i.e., actively participating in working groups in the network?	83%	Yes

Consensus was achieved if there was >75% approval. Green font and background color: Consensus achieved; Red font and background color: Consensus not achieved. * ANM: Active network management.

**Table 8:** Results of TED voting—structured patient education program according to self-management concept.

Structured Patient Education Program according to Self-Management Concept (EDU)				
Acronym *	Issue	Question	Agreement	Consensus?
EDU1	Patient education concept	Do you agree with the concept of the patient curriculum as presented? **	100%	Yes
EDU2	Contents of patient education curriculum	Do you agree that information on how to apply for a degree of disability at SHIs is given in the patient school?	100%	Yes
EDU3		Do you agree that the patient school can provide information on various social and medical aspects? ***	100%	Yes
EDU4		Do you agree that information about the development and course of the disease is given in the patient school?	100%	Yes
EDU5		Do you agree that information about the mode of action of different drugs is given in the patient school?	100%	Yes
EDU6		Do you agree that information about the side effects of different drugs is given in the patient school?	92%	Yes
EDU7		Do you agree that information about complex therapeutic procedures (deep brain stimulation, medication pump) is given in the patient school?	92%	Yes

A consensus was achieved if there was >75% approval. Green font and background color: Consensus achieved; Red font and background color: Consensus not achieved. * EDU: Structured patient education program according to self-management concept. ** Open to patients and caregivers, 7 units of 90 min each for psychoeducation lessons based on self-management approach conducted case managers or psychologists. *** On patient’s internal will to live, power of attorney, applications for rehabilitation, level of care, information on contact points, such as the medical service of the health insurance company, early retirement, information on therapy offers etc.. Electronic patient letter

## Supplementary Materials

The following are available online at https://www.mdpi.com/2077-0383/9/9/2906/s1, Table S1: Questions and items of online surveys.
