# Supplementary material for: Parkinson Network Eastern Saxony (PANOS): Reaching Consensus for a Regional Intersectoral Integrated Care Concept for Patients with Parkinson’s Disease in the Region of Eastern Saxony, Germany
Source: J Clin Med. 2020 Sep 8;9(9):2906. doi: 10.3390/jcm9092906 (PMC7563971; doi:10.3390/jcm9092906)
Supplement: Supplementary file 1 [file jcm-09-02906-s001.pdf]

Journal of *Clinical Medicine*

MDPI

# Supplementary Materials

# Parkinson Network Eastern Saxony (PANOS): Reaching Consensus for a Regional Intersectoral Integrated Care Concept for Patients with Parkinson's Disease in the Region of Eastern Saxony, Germany

**Kai F. Loewenbrück[1,2,*], Doron B. Stein[3], Volker E. Amelung[3], Robert Bitterlich[1], Martin Brumme[3], Björn Falkenburger[1,2], Annekathrin Fehre[6], Tim Feige[1], Anika Frank[1,2], Carola Gißke[5], Claudia Helmert[4], Linda Kerkemeyer[3], Andreas Knapp[4], Caroline Lang[4], Annegret Leuner[6], Carina Lummer[3], Mirella M.N. Minkman[9,10], Gabriele Müller[4], Marlena van Munster[7], Hannes Schlieter[5], Peter Themann[8], Nick Zonneveld[9,10], Martin Wolz[6]**

[1] Department of Neurology, University Hospital Dresden, 01307 Dresden, Germany; kai.loewenbrueck@uniklinikum-dresden.de
[2] German Center for Neurodegenerative Diseases (DZNE) Dresden, 01307 Dresden, Germany;
[3] Institute for Applied Health Services Research (inav), Schiffbauerdamm 12, 10117 Berlin, Germany; stein@inav-berlin.de
[4] Center for Evidence-based Healthcare, Faculty of Medicine Carl Gustav Carus, Technische Universität Dresden, Germany, caroline.lang@uniklinikum-dresden.de
[5] Chair of Wirtschaftsinformatik, Especially Systems Development, Faculty of Business and Economics, Technical University of Dresden, 01062 Dresden, Germany, bjoern.falkenburger@uniklinikum-dresden.de
[6] Department of Neurology, Elblandklinikum Meißen, 01662 Meißen, Germany; martin.wolz@elblandkliniken.de
[7] Department of Neurology, University Hospital Marburg, Marburg, Germany; munster@med.uni-marburg.de
[8] Department of Neurology, Klinik am Tharandter Wald Hetzdorf, Herzogswalder Straße 1, 09633 Halsbrücke, Germany, themann@reha-hetzdorf.de
[9] Vilans, National Centre of Expertise in Long Term Care, Utrecht, The Netherlands, m.minkman@vilans.nl
[10] Tilburg University / TIAS School for Business and Society, Tilburg, the Netherlands

* Correspondence: kai.loewenbrueck@uniklinikum-dresden.de; Tel.: +49 351 458 18518 (K.F.L)

Received: 31/07/2020; Accepted: date; Published: date

**Supplementary Materials**

**Supplementary table:**

**Table S1:** Questions and items of online surveys

**Table S1:** Questions and items of online surveys

## *General questions on demographic and care structure of the target region*

| Question | General Medicine | Neurology |
|---|---|---|
| **What medical specialty do you represent?** | 67% | 33% |

| Question | >30 | 16-30 | 5--15 | <5 |
|---|---|---|---|---|
| **How many PD patients schedule consultations with you every quarter year?** | 16% | 16% | 52% | 16% |

| Question | Metropolis | Small town | Rural area |
|---|---|---|---|
| **How would you characterize the geographic location of your practise?** | 33% | 33% | 33% |

| Question | Item | Good | Rather good | Rather bad | Bad | Don't know |
|---|---|---|---|---|---|---|
| **How would you rate care for PD patients in your region?** | Care by GPs | 24% | 42% | 24% | 0% | 9% |
| | Care by outpatient specialists | 24% | 39% | 18% | 18% | 0% |

| Question | Item | Fully agree | Agree | Moderately agree | Do not agree | Don't know |
|---|---|---|---|---|---|---|
| **Which statement on the care of PD patients would you agree with?** | Care is predominantly provided in accordance with guidelines | 12% | 61% | 6% | 6% | 15% |
| | Timely referral to specialists | 15% | 45% | 27% | 6% | 6% |
| | Patients are treated intersectorally | 6% | 33% | 27% | 15% | 18% |
| | Patients receive individually required treatment | 9% | 45% | 27% | 9% | 9% |
| | PD patients often receive insufficient or incorrect care | 6% | 33% | 33% | 12% | 12% |

| Question | Item | Very large | Large | Moderate | None | Don't know |
|---|---|---|---|---|---|---|
| **What challenges do you perceive in daily usual care reality?** | Barriers regarding timely access to specialists | 18% | 30% | 36% | 9% | 6% |
| | Insufficient education and / or insufficient sensitation of nursing staff | 12% | 33% | 36% | 9% | 9% |
| | Lack of structured support material (specific and updated information and recommendations for the treatment of PD patients) | 6% | 27% | 36% | 15% | 15% |
| | Unstructured coordination of treatment | 6% | 27% | 33% | 21% | 9% |
| | Lack of communication, resp. lack of exchange with other physicians and therapists | 6% | 39% | 30% | 15% | 6% |
| | Lack of digital components in the care concept (EPR, Apps, etc.) | 9% | 39% | 9% | 21% | 18% |
| | Lack of telemedical approaches in usual care | 6% | 30% | 15% | 24% | 21% |

## *Motivation of participants*

| Question | Item | Very much | Much | A little bit | Not at all | Don't know |
|---|---|---|---|---|---|---|
| **How much would the following items motivate you personally to participate in the network?** | Rising demand among patients | 6% | 36% | 36% | 18% | 3% |
| | Availability of a structured care pathway | 27% | 48% | 9% | 12% | 3% |
| | Support by case managers | 6% | 42% | 24% | 24% | 0% |
| | Adequate offer of therapies | 24% | 55% | 9% | 12% | 0% |
| | Advanced training curricula (for doctors, therapists and nurses) | 9% | 45% | 30% | 6% | 3% |
| | Education curricula for patients and their relatives | 9% | 58% | 12% | 15% | 6% |
| | Networking with other healthcare service providers | 27% | 42% | 12% | 15% | 0% |
| | Electronic Health Record | 6% | 15% | 30% | 39% | 6% |
| | Availability of the results of repetitive patient self-monitoring | 3% | 30% | 30% | 18% | 15% |

| | | | | | | |
|---|---|---|---|---|---|---|
| | Telemedical offers | 3% | 9% | 36% | 30% | 18% |
| | Adequate reimbursement | 12% | 33% | 36% | 12% | 6% |
| | Adequate (human and technical) resources | 18% | 45% | 27% | 6% | 3% |

## *Importance of different care aspects*

| Question | Item | 5 (relevant) | 4 | 3 | 2 | 1 (irrelevant) | Cannot judge | No answer |
|---|---|---|---|---|---|---|---|---|
| **Which of the following dimensions do you consider to be generally relevant for PD treatment?** | General health related quality of life | 59% | 18% | 18% | 0% | 0% | 0% | 0% |
| | PD-specific quality of life | 64% | 23% | 9% | 0% | 0% | 0% | 0% |
| | Motor function (e.g. gait insecurity, falls, voice)' | 64% | 27% | 5% | 0% | 0% | 0% | 0% |
| | Non-motor function (e.g. sleep, sexual function)' | 18% | 41% | 36% | 0% | 0% | 0% | 0% |
| | Cognitive function (e.g. memory, weak concentration, perceptual disorder) | 32% | 50% | 14% | 0% | 0% | 0% | 0% |
| | Emotional function (e.g. depression, anxiety, mood, impulse control disorder) | 41% | 41% | 14% | 0% | 0% | 0% | 0% |
| | Social function (e.g. aggression, social participation) | 32% | 36% | 27% | 0% | 0% | 0% | 0% |
| | Role function (e.g. occupation, parental role) | 18% | 23% | 50% | 5% | 0% | 0% | 0% |
| | Pain | 45% | 41% | 5% | 5% | 0% | 0% | 0% |
| | Adverse drug events | 41% | 36% | 14% | 5% | 0% | 0% | 0% |
| **How important do you rate the following general contents of PANOS?** | Performance of a social and nursing assessment by the case manager twice a year | | | 0% | 10% | 70% | 20% | 0% |
| | Home visit (after registration to PANOS) by the case manager to patients/their family | | | 0% | 20% | 50% | 30% | 0% |
| | Coordination and management of patients' appointments with GPs and neurologists by the case manager | | | 0% | 0% | 50% | 30% | 20% |
| | Ability for the GPs and neurologists to request services from case managers via the electronic PANOS platform | | | 10% | 40% | 50% | 0% | 0% |
| | Performance of tests (e.g. MOCA, UPDRS, etc.) by case managers | | | 10% | 10% | 60% | 20% | 0% |
| | Having a permanent personal contact person in the case managers regarding GPs' and neurologists' collaboration in PANOS | | | 30% | 20% | 30% | 0% | 0% |
| | Regular surveys among GPs and neurologists by case managers on the satisfaction of cooperation within PANOS | | | 20% | 10% | 20% | 30% | 10% |
| | Possibility of the case manager being the central contact person for patients and relatives | | | 0% | 20% | 50% | 10% | 10% |
| **Question** | **Item** | **5 (willing)** | **4** | **3** | **2** | **1 (not willing at all)** | **Cannot judge** | **No answer** |
| **Which of the following dimensions would you be willing to survey and record in a structured way in the context of your own work?** | General health related quality of life | 36% | 18% | 23% | 5% | 5% | 9% | 5% |
| | PD-specific quality of life | 36% | 41% | 9% | 5% | 0% | 5% | 5% |
| | Motor function (e.g. gait insecurity, falls, voice)' | 50% | 18% | 18% | 5% | 0% | 5% | 5% |
| | Non-motor function (e.g. sleep, sexual function)' | 32% | 23% | 18% | 18% | 0% | 5% | 5% |
| | Emotional function (e.g. depression, anxiety, mood, impulse control disorder) | 27% | 41% | 14% | 5% | 5% | 5% | 5% |
| | Social function (e.g. aggression, social participation) | 27% | 23% | 23% | 14% | 5% | 5% | 5% |

| | | | | | | | | |
|---|---|---|---|---|---|---|---|---|
| | Role function (e.g. occupation, parental role) | 18% | 18% | 27% | 23% | 5% | 5% | 5% |
| | Pain | 36% | 32% | 14% | 9% | 0% | 5% | 5% |
| | Adverse drug events | 41% | 32% | 14% | 5% | 0% | 5% | 5% |

## *Electronic Health Record*

| Question | Item | essential | Im-por-tant | optional | not impor-tant | irrelevant | Cannot judge | No answer |
|---|---|---|---|---|---|---|---|---|
| **What information about PD patients should be provided in the EHR?** | Medication plan | 82% | 14% | 0% | 0% | 0% | 0% | 5% |
| | Diagnostic reports (e.g. discharge letters, neuro-imaging-reports) | 41% | 50% | 5% | 0% | 0% | 0% | 5% |
| | Medical history reports (e.g. comorbidities, allergies, family) | 50% | 36% | 9% | 0% | 0% | 0% | 5% |
| | Social history and care situation at home (e.g. problems at home, long-term care level) | 32% | 50% | 14% | 0% | 0% | 0% | 5% |
| | Vital signs | 5% | 55% | 32% | 5% | 0% | 0% | 5% |
| | Laboratory results | 9% | 45% | 41% | 0% | 0% | 0% | 5% |
| | Clinical scales / scores | 9% | 36% | 45% | 5% | 0% | 0% | 5% |
| | Structured self-assessment questionnaires (e.g. BDI-II or UPDRS)' | 0% | 41% | 50% | 5% | 0% | 0% | 5% |
| | Other contents | 5% | 9% | 23% | 9% | 9% | 0% | 45% |
| **What functions should be provided in the EHR?** | Platform Function: Interactive Calendar for scheduling appointments | 14% | 27% | 45% | 0% | 9% | 0% | 5% |
| | Platform Function: Secured internal messaging system | 23% | 55% | 18% | 0% | 0% | 0% | 5% |
| | Platform Function: DICOM-Viewer for radiological and nuclear medical image data | 0% | 18% | 68% | 9% | 0% | 0% | 5% |
| | Platform Function: Electronic interface for the use of clinical scales/scores | 5% | 59% | 27% | 5% | 0% | 0% | 5% |
| | Platform Function: Other | 0% | 0% | 23% | 9% | 5% | 0% | 64% |
| **Question** | **Item** | **5 (Always)** | **4** | **3** | **2** | **1 (Never)** | **Cannot judge** | **No answer** |
| **Have you ever used tools for structured reporting in the context of PD?'** | | 0% | 14% | 14% | 5% | 64% | 0% | 5% |
| **Can you imagine using tools for structured reporting in the context of PD in the future?** | | 18% | 59% | 9% | 9% | 0% | 0% | 5% |

## *Patient self-management education program*

| Question | Item | Extremely important | Very impor-tant | Moderately important | Little impor-tant | Not impor-tant |
|---|---|---|---|---|---|---|
| **How important do you rate the following contents of a structured patient self-management education program?** | Knowledge about adverse drug events | 30% | 50% | 20% | 0% | 0% |
| | Knowledge about drug mechanisms and effects | 20% | 60% | 20% | 0% | 0% |
| | Strengthening of well-being | 20% | 50% | 30% | 0% | 0% |
| | Coping with anxiety | 20% | 40% | 40% | 0% | 0% |
| | Coping with depression | 20% | 40% | 40% | 0% | 0% |
| | Promotion of social support | 20% | 40% | 40% | 0% | 0% |
| | Strengthening of social competence | 20% | 10% | 70% | 0% | 0% |
| | Knowledge about the origin of the disease | 10% | 50% | 40% | 0% | 0% |
| | Coping with stress | 10% | 40% | 50% | 0% | 0% |
| | Knowledge about complex therapies (deep brain stimulation, medication pump) | 10% | 30% | 60% | 0% | 0% |
| | Ability of self-observation and introspection | 0% | 80% | 20% | 0% | 0% |

| | | | | | | |
|---|---|---|---|---|---|---|
| **How important are the following goals of the structured patient self-management education program for the referral of a patient** | Improvement of physician-patient communication | 22% | 22% | 11% | 33% | 11% |
| | Strengthening of health-promoting behavior | 22% | 22% | 55% | 0% | 0% |
| | Improvement of emotional well-being | 11% | 22% | 44% | 22% | 0% |
| | Relief of physical symptoms | 33% | 33% | 33% | 0% | 0% |
| **How important do you find the availability of a separate training session for caregiving relatives?** | | 30% | 60% | 10% | 0% | 0% |

## *Structured professional continuous education program*

| Question | Item | Annualy | Twice per year | Every quarter | No answer |
|---|---|---|---|---|---|
| **How often should Parkinson-specific training courses be offered as part of PANOS?** | | 27% | 59% | 9% | 5% |

| Question | Item | Extremely important | Very important | Moderately important | Little important | Not important |
|---|---|---|---|---|---|---|
| **How important do you rate the following contents of a structured professional continuous education curriculum?** | General PANOS program structure | 20% | 0% | 40% | 30% | 10% |
| | PANOS integrated care pathway | 20% | 10% | 40% | 30% | 0% |
| | PANOS case managers | 20% | 30% | 20% | 20% | 10% |
| | Structured patient self-management education program | 30% | 20% | 40% | 10% | 0% |
| | Structured education program for relatives | 20% | 30% | 30% | 20% | 0% |
| | Electronic Health Record | 20% | 10% | 30% | 40% | 0% |
| | Data security | 40% | 10% | 20% | 20% | 10% |
| | Care structures | 20% | 30% | 20% | 30% | 0% |
| | General case management | 10% | 10% | 60% | 20% | 0% |
| | General medical aspects of PD | 10% | 50% | 30% | 10% | 0% |
| | Societal impact of PD | 10% | 10% | 50% | 20% | 10% |
| | Epidemiology | 0% | 20% | 50% | 30% | 0% |
| | Neurobiology | 0% | 30% | 60% | 10% | 0% |
| | Genetics | 0% | 50% | 40% | 10% | 0% |
| | Diagnostics | 10% | 40% | 40% | 10% | 0% |
| | Therapy | 20% | 50% | 20% | 10% | 0% |
| | Drug therapy | 20% | 30% | 40% | 10% | 0% |
| | Adverse events & Drug interaction | 20% | 10% | 60% | 10% | 0% |
| | Sensor technology for use with PD | 0% | 20% | 50% | 30% | 0% |
| | Rehabilitation programs for PD patients | 10% | 50% | 30% | 10% | 0% |
| | General comorbidities | 0% | 40% | 50% | 10% | 0% |
| | Internistic comorbidities | 10% | 70% | 10% | 10% | 0% |
| | Psychiatric comorbidities | 10% | 40% | 40% | 10% | 0% |
| | Psychosocial support | 10% | 40% | 40% | 10% | 0% |
| | Psychotherapy | 10% | 10% | 30% | 40% | 0% |
| | Sociotherapy | 10% | 20% | 50% | 20% | 0% |
| | Self-help | 20% | 10% | 60% | 10% | 0% |
| | Occupational therapy | 20% | 50% | 20% | 10% | 0% |
| | Physiotherapy | 30% | 40% | 20% | 10% | 0% |
| | Speech therapy | 30% | 40% | 20% | 10% | 0% |
| | Foster care for PD patients | 20% | 10% | 50% | 10% | 0% |
| | Motor complications | 10% | 40% | 40% | 10% | 0% |

| Question | Item | Selected by |
|---|---|---|
| **What are your preferred training formats (multiple selection possible)?** | Personal information events with workshop character | 40% |
| | Online training, E-learning formats (self-study courses) | 40% |
| | Online trainings (live with speakers) | 50% |
| | Classroom training | 60% |
| | Case studies | 50% |
| | Advanced trainings during the meetings of existing regional quality circles | 20% |
| | Regional training courses in a new PANOS-specific format | 50% |
| | Central training courses (e.g. in PD centers) | 40% |

## *Information on the program*

| Question | Item | Selected by |
|---|---|---|
| **How would you like to be informed about current developments in the project (multiple selection possible)?** | Collaborative platforms like Confluence | 0% |
| | Print-Newsletters | 20% |
| | PANOS-Website | 40% |
| | Regular information events | 50% |
| | E-Mail-Newsletters | 80% |

## *Miscellaneous*

| Question | Item | Yes | No | Cannot judge | No answer |
|---|---|---|---|---|---|
| **Did you take part in the 1st PANOS Workshop on 29.01.2020?** | | 55% | 45% | 0% | 0 |
| **Are you registered for the 2nd PANOS Workshop (11.03.2020) or do you plan to participate?** | | 68% | 27% | 0% | 5% |
| **Are you registered for the 3rd PANOS Workshop (22.04.2020) or do you plan to participate?** | | 68% | 18% | 0% | 14% |
| **Do you consider it sufficient to be able to contact a specialist via the internal messaging system of the PANOS platform in case of emergencies or uncertainties?** | | 77% | 18% | 0% | 5% |
| **Would it be generally practicable for you if the PANOS users involved were to communicate via the internal PANOS news platform?** | | 86% | 9% | 0% | 5% |

© 2020 by the authors. Submitted for possible open access publication under the terms and conditions of the Creative Commons Attribution (CC BY) license (http://creativecommons.org/licenses/by/4.0/).
